# Improving the Efficiency of Organic Solar Cells with Methionine as Electron Transport Layer

**DOI:** 10.3390/molecules27196363

**Published:** 2022-09-27

**Authors:** Yujie Xu, Hang Zhou, Pengyi Duan, Baojie Shan, Wenjing Xu, Jian Wang, Mei Liu, Fujun Zhang, Qianqian Sun

**Affiliations:** 1 School of Physics and Electronics, Shandong Normal University, Jinan 250014, China; 2 Key Laboratory of Luminescence and Optical Information, Ministry of Education, Beijing Jiaotong University, Beijing 100044, China; 3 College of Physics and Electronic Engineering, Taishan University, Taian 271021, China

**Keywords:** organic solar cells, biological material, methionine, ZnO

## Abstract

Interface modification is an important way to get better performance from organic solar cells (OSCs). A natural biomolecular material methionine was successfully applied as the electron transport layer (ETL) to the inverted OSCs in this work. A series of optical, morphological, and electrical characterizations of thin films and devices were used to analyze the surface modification effects of methionine on zinc oxide (ZnO). The analysis results show that the surface modification of ZnO with methionine can cause significantly reduced surface defects for ZnO, optimized surface morphology of ZnO, improved compatibility between ETL and the active layer, better-matched energy levels between ETL and the acceptor, reduced interface resistance, reduced charge recombination, and enhanced charge transport and collection. The power conversion efficiency (PCE) of OSCs based on PM6:BTP-ec9 was improved to 15.34% from 14.25% by modifying ZnO with methionine. This work shows the great application potential of natural biomolecule methionine in OSCs.

## 1. Introduction

Organic solar cells (OSCs) have become the focus of new energy research worldwide due to their low cost, light weight, transparency, good flexibility, and large area preparation [1,2,3]. OSCs are moving towards commercialization with the continuous improvement of various research groups on device engineering and the synthetic tailoring of organic molecules [4,5,6,7]. The power conversion efficiency (PCE) and stability of OSCs are still the main research focus. In the traditional device structure of OSCs, the acidity of the classic hole transport layer poly(3,4-ethylenedioxythiophene): poly(styrenesulfonic acid) (PEDOT:PSS) will corrode the indium tin oxide (ITO) to a certain degree over time, which will lead to the aging of the device [8,9,10], while the metal electrodes (such as calcium [Ca] and aluminium [Al]) with low work function (WF) are unstable due to easy oxidation [11,12]. Inverted OSCs with high WF metals as anodes (such as silver [Ag] and gold [Au]) and modified ITO as cathodes have been developed to improve these problems. OSCs with inverted structures have more advantages to device stability and device preparation [13,14,15,16].

The introduction of an electron transport layer (ETL) between the ITO electrode and the active layer is an effective strategy to get better performance from the inverted OSCs [17,18]. A excellent ETL can not only adjust the energy level arrangement between the acceptor material and the electrode and promote single charge transport, but also modify the active layer morphology and regulate the built-in electric field of the device [19,20,21,22,23,24]. Due to its diverse preparation methods, high transparency, low WF, and high electron mobility, zinc oxide (ZnO) has frequently been used as an ETL material in inverted OSCs [25,26,27,28]. However, ZnO film prepared by the sol-gel method has some disadvantages such as uneven surface morphology, poor contact with the active layer, and high surface defect density [29,30,31,32]. Among them, the uneven surface morphology of ZnO film is not conducive to the film-forming of the active layer on its surface. The surface defects of ZnO film will affect the transfer and recombination process of charge. The bad contact between ZnO and the active layer will cause larger series resistance of the device. These problems can limit the application of ZnO as ETL on the inverted OSCs. To reduce the impact of these issues, materials such as polymers, polyelectrolytes, small molecules, ionic liquids, and self-assembled monolayers (SAMs) have been applied to the surface modification of ZnO [32,33,34,35]. Organic materials account for most of these, which generally have complex synthetic procedures or large gaps between batches, which is not conducive to the practical application of the OSCs [36,37,38]. Therefore, the development of new and efficient ETL materials is very important. Natural biological materials are one of the potential ETL candidates. Abundant natural biological materials are widely found in animals, plants, and microorganisms in nature. Simultaneously, natural biological materials have the characteristics of being environmentally friendly and having diverse functional groups. Some natural biological materials have been applied to the interface modification of OSCs, such as deoxyribonucleic acid (DNA), amino acids, and cellulose. Brown et al. modified ZnO with DNA to improve the rectification ratio and shunt resistance of the cells as well as reduce the WF of the electrode [39]. Ahmad et al. observed appropriate WF and surface wettability with homogenous surface morphology by using polyaspartic acid to modify ZnO [40]. Wu et al. used cellulose and its derivatives as the ETL of OSCs, which improved light absorption and interface contact, thus improving the device’s performance [41]. The above natural biomaterials were introduced into OSCs as ETLs, and the corresponding device performance was improved. 

**Figure 1:**
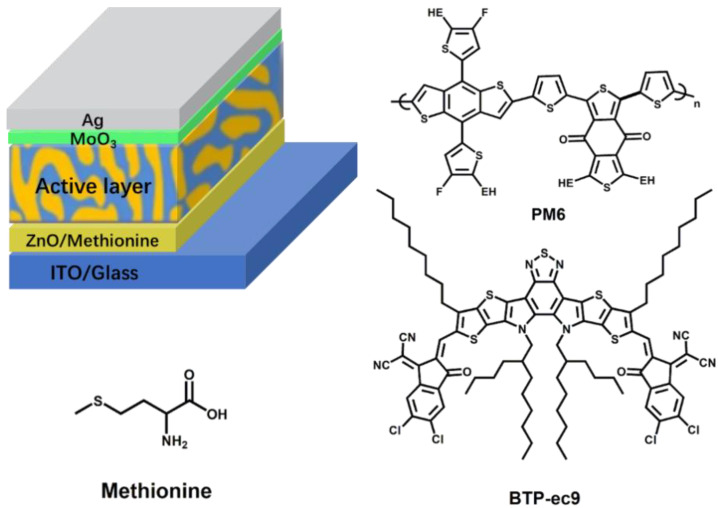
Schematic diagram of the device structure for used OSCs and the chemical structures for the used materials.

The amino acid methionine is a biological material widely present in protein-rich foods such as beans. In this work, we successfully introduced methionine as ETL into the inverted OSC. The device structure of used OSCs (ITO/ETL/PM6: BTP-ec9/MoO_3_/Ag) and the chemical structures of used materials (PM6, BTP-ec9, methionine) are shown in Figure 1. The PCEs of the OSCs are increased from 5.45% to 10.33% by adding methionine on the surface of ITO, with enhanced the short-circuit current density (*J_SC_*) of 24.43 mA cm^−2^, open circuit voltage (*V_OC_*) of 0.67 V and fill factor (FF) of 63.16%. The PCE of OSCs based on PM6:BTP-ec9 was improved to 15.34% from 14.25% by modifying ZnO with methionine, resulting from the synchronously enhanced *J_SC_* of 25.52 mA cm^−2^, FF of 71.58%, and *V_OC_* of 0.84 V. The analyses based on optical characterization indicate that the modification of methionine on the surface of ZnO film cannot absorb or block the sunlight irradiated from the transparent electrode into the active layer and can significantly reduce the surface defects of ZnO. The analyses of the morphological characteristics of thin films show that the modification of methionine for the ZnO surface can optimize the surface morphology of ZnO and can improve the compatibility between ZnO and the active layer. The analyses based on the electrical characteristics of thin films and the devices indicate that the modification of methionine can significantly improve energy level matching between ETL and the acceptor, reduce interface resistance, reduce charge recombination, and promote charge transport and charge collection.

## 2. Results and Discussion

**Figure 2:**
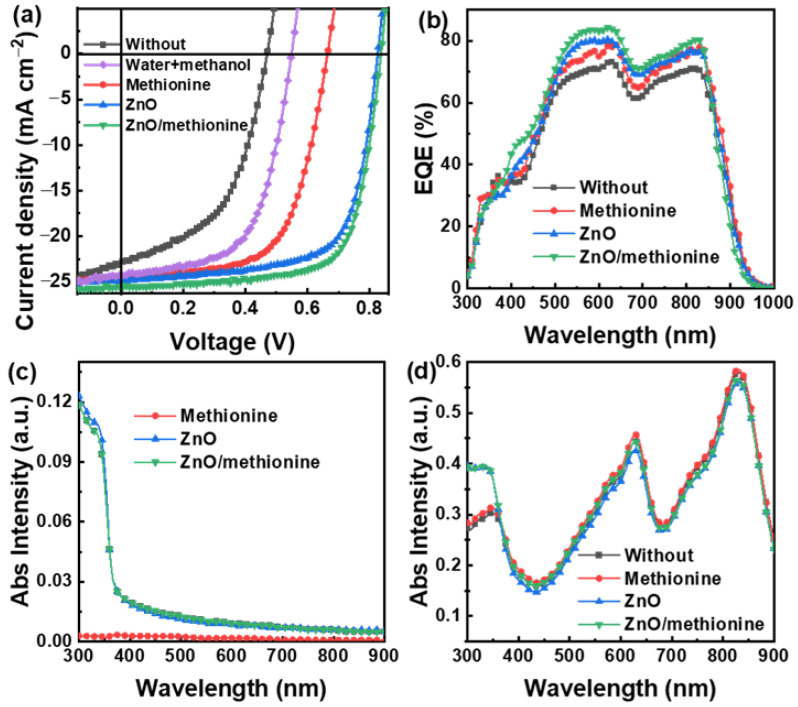
(**a**) *J-V* characteristic curves and (**b**) EQE spectra of OSCs with different ETLs. (**c**) Absorption spectra of different ETLs films and (**d**) different ETLs/active layer films.

In this work, the inverted OSCs with the structure of ITO/ETL/PM6:BTP-ec9/MoO_3_/Ag were prepared to study the effect of methionine. The current density and voltage (*J-V*) curves of OSCs were obtained under AM 1.5 G simulated sunlight of 100 mW cm^−2^, as shown in Figure 2a. The key photovoltaic parameters of *J_SC_*, *V_OC_*, FF, and PCE are summarized in Table 1 for OSCs with different ETLs. The *V_OC_*, *J_SC_*, and FF of the OSCs without ETL are 0.47 V, 23.25 mA cm^−2^, and 49.88%, respectively, which leads to a low PCE of 5.45%. The OSCs based on methionine as ETL exhibit a PCE of 10.33%, a *V*_OC_ of 0.67 V, a *J*_SC_ of 24.43 mA cm^−2^, and a FF of 63.16%. In this work, a mixed solution was used as the solvent of methionine. The weight ratio of deionized water and methanol is 1:9, and the optimized concentration of methionine solution was 1 mg mL^−1^. The *J-V* Characteristic curves of OSCs with methionine as ETL under different concentrations (0.5 mg mL^−1^, 1 mg mL^−1^, 2 mg mL^−1^) of methionine solution are shown in Figure S1 and Table S1. The PCE, *J_SC_*, and FF of OSCs with methionine as ETL under a methionine solution concentration of 1 mg mL^−1^ are improved when compared with OSCs with methionine as ETL under at a concentration of 0.5 mg mL^−1^. When the concentration is further increased, the PCE of OSCs is reduced along with the increased FF and the reduced *J_SC_*, because of which it may be said that an overly thick methionine layer leads to large series resistance. Previous studies have proved that methanol can also be applied to embellish the ETL of OSCs [42]. Therefore, OSCs with mixed solvents used to modify ITO were prepared as a contrast device. The OSCs with mixed solvents for the modification ITO exhibit a *J_SC_* of 24.16 mA cm^−2^, a FF of 60.40%, and a *V_OC_* of 0.55 V, resulting in a PCE of 8.02%. The contact potential difference (CPD) of the ITO modified by different ETLs was obtained by Kelvin probe force microscopy (KPFM). The obtained CPD image, CPD data, and WF are shown in Figure S2 and Table S2, where 4.70 eV is the WF of the determined ITO, which was used to aid in the calculation of other WF. The WF of the ITO modified by mixed solvent was decreased from 4.70 eV to 4.54 eV, which is attributed to the interface dipole between ITO and active layer induced by methanol [42]. The introduction of interface dipoles may increase the built-in potential of the device, which is conducive to charge transmission and collection. The performance of OSCs with methionine as their ETL has been improved to a greater extent than that of the OSCs with mixed solvent as ETL, indicating that the solute methionine in the methionine solution plays a major role in cathode interface modification [43]. A PCE of 14.25% was acquired in the OSCs with ZnO as the ETL, along with a *J_SC_* of 24.79 mA cm^−2^, a *V_OC_* of 0.83 V, and a FF of 69.26%. An optimized PCE of 15.34% was achieved in OSCs with ZnO/methionine as their ETLs, along with a *J_SC_* of 25.52 mA cm^−2^, a *V_OC_* of 0.84 V, and a FF of 71.58%. The *J*_SC_, *V*_OC_, and FF of OSCs were increased by the modification of ZnO with methionine, resulting in an increased PCE. The above results show that whether methionine is used as ETL alone or as a double ETL with ZnO, the performance of OSCs is improved.

A series of device and film characteristics were measured to further explore the working mechanisms of methionine at the cathode interface. The external quantum efficiency (EQE) spectra for OSCs with different ETLs are shown in Figure 2b. The EQE of the OSCs with methionine, ZnO, or ZnO/methionine as ETLs are larger than that of the OSCs without ETL in the spectral range of 400–850 nm. For different devices, the changing trend of EQE conforms to the law of corresponding *J*_SC_. Among them, the EQE of the OSCs based on ZnO/methionine as ETLs has the greatest enhancement effect, which may be attributed to multiple factors, such as the optimized morphology of the active layer, the better ohmic contact of active layer and cathode, and the improved compatibility between ETL and the active layer. In the wavelength range of 300–400 nm, the EQE enhancement effect of OSCs with methionine as their ETL is greater than that of OSCs based on ZnO or ZnO/methionine as their ETL. The absorption spectra and transmission spectra of ZnO, methionine, and ZnO/methionine films with quartz glass as a substrate were obtained in order to study the light transmittance of ETLs, as shown in Figure 2c and Figure S3a. The values of the absorption and transmission spectra of methionine film approach 0 and 100% in the wavelength range of 300–900 nm, respectively. This result indicates the sunlight irradiated from the transparent electrode into the active layer will not be blocked by the methionine film. The absorption or transmission spectra of ZnO or ZnO/methionine films coincide in the wavelength range of 300–900 nm, which confirms the high light transmission performance of methionine films again. Similar absorption intensities in the range of 300–900 nm are obtained in the ZnO and ZnO/methionine films, which should be attributed to the self-absorption of ZnO. The self-absorption of ZnO ought to be the principal consideration for the larger EQE value of OSCs with methionine as ETL in the wavelength range of 300–400 nm. The light absorption of the active layer in OSCs with different ETLs is explored by the absorption and transmission spectra of the active layer and ETLs/active layer, as shown in Figure 2d and Figure S3b. The absorption intensities of the ZnO/active layer films and ZnO/methionine/active layer films are significantly enhanced compared to those of the active layer only in the range of 300–400 nm, which should be due to the self-absorption of ZnO. The absorption and transmission spectra of the methionine/active layer films and active layer films are almost identical. These results further confirm that the sunlight irradiated from the transparent electrode into the active layer cannot be absorbed or blocked when methionine is an ETL of OSCs.

**Figure 3:**
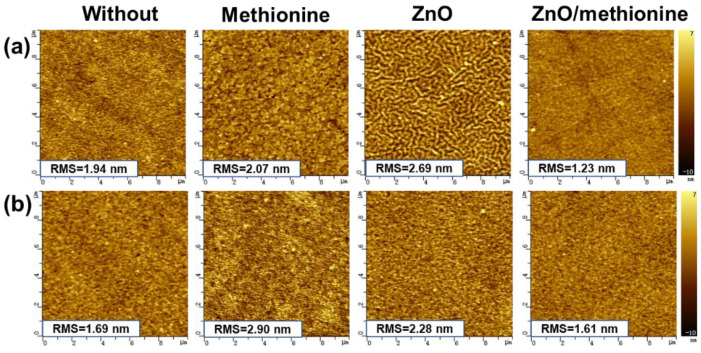
(**a**) AFM height images of ITO/ETL and (**b**) ITO/ETL/active layer films.

**Figure 4:**
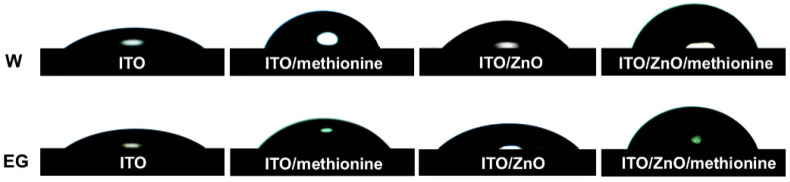
Contact angle images of ITO/ETLs with deionized water or ethylene glycol as the probing liquids.

The surface morphology of different ETL films was studied by atomic force microscopy (AFM). The measured height images are given in Figure 3a. The relatively smooth surface was obtained with a similar root–mean–square (RMS) surface roughness of 1.94 nm and 2.07 nm for ITO and ITO/methionine films, respectively. A relatively rough surface with an RMS surface roughness of 2.69 nm was formed on the surface of ITO/ZnO due to the formation of the corrugated micro-nano structure. The RMS surface roughness decreased from 2.69 nm to 1.23 nm when methionine was used to modify the surface of ZnO. The smoother and more orderly surface morphology may be formed because methionine can fill or cover the gap in the uneven surface of the ITO/ZnO film [44]. Compared with the original ITO/ZnO film, ITO/ZnO/methionine film may form a better interface contact with the active layer, resulting in better electron transport and promoting the improvement of *J_SC_* and FF [45,46]. The surface morphology of active layers (PM6:BTP-ec9) prepared on the surface of ITO, ITO/methionine, ITO/ZnO, and ITO/ZnO/methionine was characterized by AFM, as shown in Figure 3b. Some small aggregation is observed on the surface of ITO/methionine/PM6:BTP-ec9 film. A relatively smooth surface with an RMS surface roughness of 1.69 nm and 2.28 nm is observed on the surface of ITO/PM6:BTP-ec9 and ITO/ZnO/PM6:BTP-ec9, respectively. The surface of the ITO/ZnO/methionine/PM6:BTP-ec9 film has the most uniform morphology with an RMS surface roughness of 1.61 nm, which means that the PM6:BTP-ec9 film may have better interface contact with the ITO/ZnO/methionine. The more obvious difference in surface height for the active layer can be seen in the line scanning diagram and height histogram as shown in Figure S4. The different surface morphology of the active layer may be related to the surface energy of ETLs [47]. The contact angles of different ETLs with deionized water (W) and ethylene glycol (EG) as detection liquids were measured to verify or refute this hypothesis, as shown in Figure 4. The surface energy of the film was calculated by the contact angle measurement method combined with the Wu model, as shown in Table 2 [48]. According to the contact angle calculation, the surface energies of ITO, ITO/methionine, ITO/ZnO, and ITO/ZnO/methionine films are 63.76, 39.07, 51.65, and 36.09 mN m^−1^, respectively. The surface energies of ITO/methionine and ITO/ZnO/methionine are lower than that of ITO and ITO/ZnO. The different surface energies of ETLs may lead to different wettability, which may affect the drying kinetics of the active layer spin-coated in ETLs, thereby affecting the morphology of the active layer [47]. From the water contact angle diagram it can be seen that the water contact angle of ITO/ZnO/methionine film is significantly larger than that of ITO/ZnO film, which indicates that the surface of ZnO becomes more hydrophobic after methionine modification. The more hydrophobic surface of ETL will enhance the close contact between ETL and the active layer, thus improving the atmosphere stability of OSCs [49].

**Figure 5:**
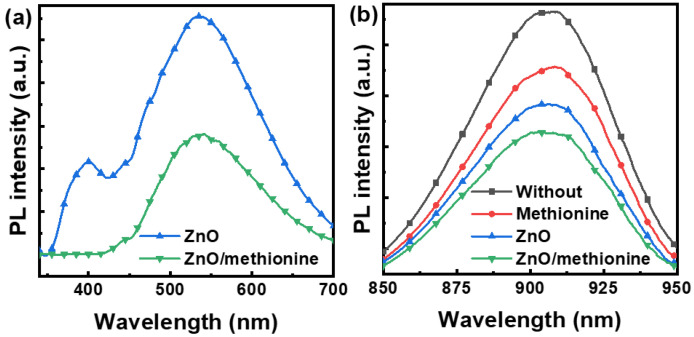
PL spectra of different (**a**) ETL films and (**b**) ITO/ETL/active layer films.

The effect of methionine on the electron extraction behavior of ZnO film was further studied by the measurement of photoluminescence (PL) spectra, as shown in Figure 5a. Under the effect of a 320 nm excitation light, the ZnO film shows two emission peaks centered at 530 nm and 400 nm. The emission peak at 400 nm comes from free exciton recombination at the near band edge [50]. The emission peak at 530 nm stems from the surface defect of the oxygen vacancy [17,51]. The oxygen vacancy can become a potential electron well, and each vacancy can capture one or two electrons, thereby increasing the probability of charge recombination [52]. The intensity of the PL spectra for ZnO/methionine films showed a significant reduction compared with that of the ZnO films. The decreased emission intensity of ZnO/methionine film at 400 nm may originate from the decreased band edge emission. The decreased emission intensity of ZnO/methionine films at 530 nm may originate from the reduction of oxygen defects, which may be conducive to improving the charge extraction efficiency. The above phenomena show that the modification of methionine on the surface of ZnO film can significantly reduce the surface defects of ZnO. The photoinduced charge transport behavior in the active layer can also be detected by PL spectra. The PL spectra for active layer film prepared on the surface of different ETLs and separate active layer film under the same conditions are shown in Figure 5b. The active layer films spin-coated in the different ETLs have greater PL quenching efficiencies compared with the separate active layer film. Higher PL quenching efficiency indicates that more efficient charge transport and less charge recombination loss can be realized in the active layer, which is conducive to obtaining higher photocurrent in the devices [53,54]. The PL quenching degree of the ZnO/methionine/active layer is the largest, indicating that the surface modification of ZnO by methionine is conducive to promoting electron transfer and reducing charge recombination.

In order to more deeply understand the influence of different ETLs on charge transport, the charge mobility was fitted by the space charge limited current (SCLC) model. Under dark state conditions, the typical log (*J*) and log (*V*) curves were obtained for the pure hole device (ITO/PEDOT:PSS/PM6:BTP-ec9/MoO_3_/Ag) and pure electron devices (ITO/ETLs/PM6: BTP-ec9/PDIN/Al), as shown in Figure S5. The slopes of the log (*J*) and log (*V*) curves of these devices are close to 1 at low applied voltage, which signifies that the interface contact in the device can be viewed as an ohmic contact. At higher applied voltages, the slope of the log (*J*) and log (*V*) curves is about 2, which indicates that the injection rate of the carriers exceeds the recombination rate, thus forming SCLC flow [55,56]. The corresponding curves of ln(*Jd*^3^/*V*^2^)-(*V*/*d*)^0.5^ for the pure hole device and pure electronic devices with different ETLs are shown in Figure S6. The calculated hole mobility (*μ_h_*) is 1.02 × 10^−3^ cm^2^ V^−1^s^−1^. The calculated electron mobility (*μ_e_*) and *μ_h_*/*μ_e_* ratio of the active layer are listed in Table S3. The *μ_e_* of the active layer for the device without ETL is 4.69 × 10^−4^ cm^2^ V^−1^ s^−1^. The *μ_e_* of the active layer for the device with methionine or ZnO as ETL is increased to 5.72 × 10^−4^ cm^2^ V^−1^ s^−1^ and 6.33 × 10^−4^ cm^2^ V^−1^ s^−1^. The *μ_e_* of the active layer for the device with ZnO/methionine as ETLs is further increased to 1.27 × 10^−3^ cm^2^ V^−1^s^−1^. These data mean that the modification of methionine on the surface of ITO or ITO/ZnO is conducive to promoting electron transport. The *μ_h_*/*μ_e_* ratio of the active layer for the device without ETL is 2.18. The *μ_h_*/*μ_e_* ratios of the active layers of devices with methionine, ZnO, or ZnO/methionine as ETLs are 1.79, 1.62, and 0.80, respectively. These data show that the modification of methionine as ETL on the surface of ITO or ITO/ZnO can promote the transport balance of electrons and holes in the OSCs.

**Figure 6:**
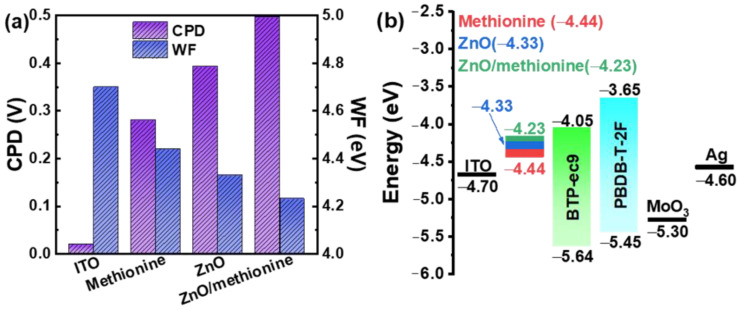
(**a**) WFs and CPDs of ITO modified by different ETL films. (**b**) Simplified energy levels diagram of OSCs with PM6:BTP-ec9 as the active layers.

To achieve effective electron extraction in OSCs, the match between the WF of ETL and the lowest unoccupied molecular orbital (LUMO) of the acceptor is a prerequisite. The CPDs of ITO/ETLs were measured by the KPFM method in order to explore the degree of the energy level matching between ETL and the active layer. The obtained CPD data and image are shown in Figure S1. The WFs are calculated by the particular WF of the ITO (4.70 eV). The WFs and CPDs are shown in Table S2 and Figure 6a for ITO/different ETLs films. Compared with ITO, the WF of ITO/methionine, ITO/ZnO, and ITO/methionine/ZnO are lowered to 4.44 eV, 4.33 eV, and 4.23 eV, respectively. The schematic diagram of the energy levels is shown in Figure 6b. The ITO modified with ZnO/methionine has the lowest WF value, which better matches the energy level of the LUMO level of the acceptor. Meanwhile, the decreased WFs of ITO/ETLs can lead to enhanced built-in potential and increased *V_OC_*. Appropriate energy level matching and increased built-in potential can improve electron transport and collection while reducing the series resistance of OSCs [57].

**Figure 7:**
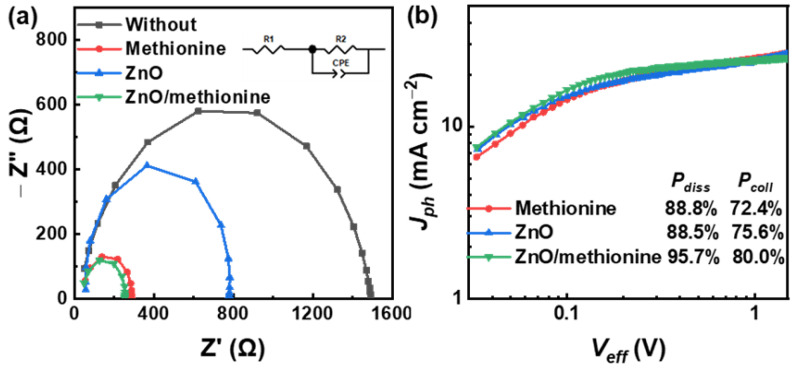
(**a**) Nyquist plots of the OSCs without ETL, with methionine, ZnO, or ZnO/methionine as ETLs. (**b**) *J_ph_*–*V_eff_* curves of OSCs with methionine, ZnO, or ZnO/methionine as ETLs.

To further explore the charge transport characteristics of the device, electrochemical impedance spectroscopy (EIS) was performed in a specific frequency range (1 Hz–1 MHz) under the application of *V_OC_* bias. Figure 7a shows the Nyquist plots of OSCs obtained by EIS. According to the inserted equivalent circuit diagram, the series resistance (*R*_1_) is related to the sheet resistance of the electrodes and electrical contacts, which can be explained by the initiation point of the Nyquist diagram. Four OSCs based on different ETLs showed small and similar *R*_1_ values. The charge transfer resistance (*R*_2_) is related to the interface resistance of the electrode/active layer and the internal resistance of the active layer, which could be described by the semicircle radius of the Nyquist diagram [58,59]. The OSC without ETL shows a relatively large *R*_2_ value, which may cause greater charge transport blockage. The *R*_2_ values are decreased for the OSCs based on methionine, ZnO, or ZnO/methionine as ETLs. The OSCs with ZnO/methionine as ETLs have the lowest *R*_2_, which could be attributed to the smooth surface morphology of the active layer and the good compatibility between ETLs and the active layer. The lower *R*_2_ value can promote charge transport and charge collection, resulting in a higher FF [60]. The photocurrent density’s (*J_ph_*) dependence on the effective voltage (*V_eff_*) of OSCs was investigated in order to further explore the influence of ETLs on the exciton dissociation and charge collection, which is shown in Figure 7b [61], where *J_ph_* equals the value of current density under illumination (*J_L_*) minus current density in the dark (*J_D_*), and *V_eff_* equals the value of the voltage at *J_ph_* = 0 (*V_0_*) minus the applied voltage in the devices (*V*). When *V_eff_* reaches 1.5 V, the photocurrent density represents the saturation photocurrent density (*J_sat_*). Under a short circuit, the photocurrent density represents *J_pha_*. The exciton dissociation efficiency (*P_diss_*) could be reckoned with by *J_pha_/J_sat_*. Under maximum output power, the photocurrent density represents *J_phb_*. The charge collection efficiency (*P_coll_*) can be reckoned with by *J_phb_/J_sat_*. The *P_diss_* and *P_coll_* are 95.7% and 80.0% for the optimized OSCs with ZnO/methionine as ETLs, which are larger than those of 88.8% and 72.4% for the methionine-based OSCs as well as bigger than those of 88.5% and 75.6% for the ZnO-based OSCs. These results show that OSCs with ZnO/methionine as ETL show better charge collection and exciton dissociation efficiency, resulting in the highest FF [62]. The *J_SC_* and FF of OSCs are also affected by charge recombination. Therefore, the charge recombination degree in OSCs with different ETLs was researched according to the *J–V* curve of OSCs under light intensities (*P_light_*) of 100, 79, 50, 39.5, 25, 12.5, or 10 mW cm^−2^ (Figure S7). The *J_SC_* and *V_OC_* as functions of *P_light_* are described in Figure S8a,b, respectively. The dependence of the *J_SC_* on the light intensity follows a power law (*J_SC_* ∝ (*P_light_*)^α^), where α stands for an exponential factor. When bimolecular recombination is inhibited, the exponential factor α should be close to 1, which can indicate a weak loss of charge. The α value for OSCs with ZnO is 0.950. The α value for ZnO/methionine as ETL is 0.978. This indicates that the bimolecular recombination in OSCs with ZnO/methionine as ETL is relatively lower compared with ZnO devices. The trap-assisted recombination degree in OSCs could be determined by the relationship between *V_OC_* and *P_light_* [*V_OC_* ∝ n(kT/q)ln(*P_light_*)]. Where n is the ideal factor under illumination, which represents the recombination dynamics in the OSCs, k is Boltzmann’s constant, T is absolute temperature, and q is basic charge. When it comes to trap-assisted recombination, the slope of the *V_OC_*–*P_light_* curve may be greater than kT/q. The slopes of OSCs based on ZnO/methionine as ETLs (1.172 kT/q) are smaller than those of OSCs based on ZnO as ETLs (1.426 kT/q). This indicates that the single-molecule or trap-assisted recombination of OSCs can be restrained to a certain extent by introducing methionine on the surface of ZnO. These results indicate that the introduction of methionine between ZnO and the active layer can effectively inhibit the bimolecular recombination and trap-assisted recombination, thus significantly improving the performance of devices using ZnO/methionine as ETLs. To investigate the effect of methionine on the device stability, the *J–V* curves of ZnO or ZnO/methionine-based OSCs were repeatedly measured for 35 h while stored in a high-purity nitrogen-filled glovebox. The normalized PCEs versus storage time are shown in Figure S9. After 35 h, the PCEs of the OSCs with ZnO or ZnO/methionine as ETLs were decreased to 91%, or 93% of the corresponding initial values. The OSCs with methionine or ZnO/methionine as ETLs have higher stability compared to IOSCs with ZnO as ETL. The surface modification of ZnO by methionine has some positive effects on the stability of OSCs.

## 3. Conclusions

In this work, a natural biomolecule methionine was successfully applied to the inverted OSCs. The optimized OSCs with methionine/ZnO as double ETLs exhibit a PCE of 15.34%, along with a synergistically increased *J_SC_* of 25.52 mA cm^−2^, FF of 71.58%, and *V_OC_* of 0.84 V compared with the OSCs based on ZnO as ETL. A series of optical, morphological, and electrical characterization analysis results for thin films and the devices shows the numerous advantages of the surface modification of ZnO with methionine. The surface modification of ZnO with methionine can cause significantly reduced surface defects for ZnO, optimized surface morphology of ZnO, improved compatibility between ETL and the active layer, better-matched energy levels between ETL and the acceptor, reduced interface resistance, reduced charge recombination, and enhanced charge transport and collection. This work indicates that methionine has great potential as a surface modification material to improve the performance of OSCs.

## Figures and Tables

**Table 1:** Key photovoltaic parameters of OSCs with different ETLs.

ETL	*Jsc* (mA cm^−2^)	*Voc* (V)	FF (%)	PCE (%)
Without	23.25	0.47	49.88	5.45
Water+methanol	24.16	0.55	60.40	8.02
Methionine solution	24.43	0.67	63.16	10.33
ZnO	24.79	0.83	69.26	14.25
ZnO/methionine solution	25.52	0.84	71.58	15.34

**Table 2:** Key photovoltaic parameters of OSCs with different ETLs.

ITO/ETLs	$\theta_{W}$ (^o^)	$\theta_{EG}$ (^o^)	Surface Energy
			(mN m^−1^)
Without	33.33	31.51	63.76
Methionine solution	66.67	49.15	39.07
ZnO	49.31	39.12	51.65
ZnO/methionine solution	73.10	69.50	36.09

## Supplementary Materials

The following are available online at https://www.mdpi.com/article/10.3390/molecules27196363/s1, Table S1: Key photovoltaic parameters of OSCs with methionine as ETL under different concentrations of methionine solution, Table S2: CPD and WFs of ITO modified by different ETLs, Table S3: Hole mobility and electron mobility extracted from the SCLC model, Figure S1: J-V characteristics curves of OSCs with methionine as ETL under different concentrations of methionine solution, Figure S2: (**a**) CPD data and (**b**–**f**) CPD images of ITO modified by different ETLs captured in KPFM, Figure S3: (**a**) Transmission spectra of different ETLs films and (**b**) different ETLs/active layer films, Figure S4: (**a**) Line scanning height of the AFM images of PM6:BTP-ec9 films spin-coated on ITO, ITO/methionine, ITO/ZnO, and ITO/ZnO/methionine; (**b**) surface height histogram extracted from corresponding AFM image, Figure S5: (**a**) Shows a typical plot of log (J) versus log (V) curves obtained when holes are injected into a hole-only device, and (**b**) shows the same curve when electrons are injected into a purely electronic device. The dotted line represents the slope, Figure S6: ln(JL3/V2)-(V/L)0.5 curves for the SCLC fitting of (**a**) hole-only devices with the structure of ITO/PEDOT:PSS/active layer/MoO3/Al and (**b**) electron-only devices with the structure of ITO/ETL/active layer/PDIN/Al, Figure S7: J-V curves of OSCs with ZnO or ZnO/methionine as ETLs under AM 1.5G illumination with the light intensities of 100, 79, 50, 39.5, 25, 12.5, or 10 mW cm^−2^, respectively, Figure S8: (**a**) JSC and (**b**) VOC dependence on the light intensity of OSCs with ZnO or ZnO/methionine as ETLs, Figure S9: Normalized PCE versus storage time for ZnO or ZnO/methionine-based OSCs stored in a high-purity nitrogen-filled glovebox.
