# Supplementary material for: Improving the Efficiency of Organic Solar Cells with Methionine as Electron Transport Layer
Source: Molecules. 2022 Sep 27;27(19):6363. doi: 10.3390/molecules27196363 (PMC9572969; doi:10.3390/molecules27196363)
Supplement: Supplementary file 1 [file molecules-27-06363-s001.zip › molecules-1934700-supplementary.pdf]

*molecules*

MDPI

# Improving the efficiency of inverted organic solar cells with methionine as electron transport layer

**Yujie Xu[1], Hang Zhou[1], Pengyi Duan[1], Baojie Shan[1], Wenjing Xu[2], Jian Wang[3], Mei Liu[1,*], Fujun Zhang[2,*] and Qianqian Sun[1,*]**

[1] School of Physics and Electronics, Shandong Normal University, Jinan 250014, China
[2] Key Laboratory of Luminescence and Optical Information, Ministry of Education, Beijing Jiaotong University, Beijing 100044, China
[3] College of Physics and Electronic Engineering, Taishan University, Taian 271021, China
* Correspondence: liumei@sdnu.edu.cn (M.L.); fjzhang@bjtu.edu.cn (F.Z.); qianqiansun@sdnu.edu.cn (Q.S.)

## 1. Experimental section

### 1.1. Materials

Indium tin oxide (ITO) glass substrates with a sheet resistance of 15 Ω square$^{-1}$ were acquired from South China Science & Technology Company Limited. The methionine was purchased from Sinopharm Chemical Reagent Co. Ltd. The used donor and acceptor materials (PM6 and BTP-ec9) were purchased from Organtec Ltd. The methionine was dissolved in blend deionized water and methanol solvents with a weight ratio of 1:9 at a total concentration of 1 mg ml$^{-1}$, and then the solution was continuously stirred at room temperature for 12 hours. The blend of PM6:BTP-ec9 with a weight ratio of 1:1.2 was dissolved in chloroform solvent at a total concentration of 17.6 mg ml$^{-1}$, and then the solution was continuously stirred at 40 $^{o}$C for 12 hours. After that, 0.5 vol% DIO was doped into the PM6:BTP-ec9 blend solutions.

### 1.2. Device fabrication

The ITO glass substrates were sequentially pre-cleaned in the ultrasonic baths containing detergent, deionized water, or ethanol, respectively. Then the cleaned ITO substrates were dried with nitrogen gas and then treated with oxygen plasma for 90 s to improve work function and clearance. The zinc oxide (ZnO) solution (80 µl) was spin-coated on ITO substrates at 4000 rounds per minute (rpm) for 30 s and then annealed at 150°C for 30 min in atmospheric air. The methionine solution (80 µl) was spin-coated on ITO substrates with ZnO film at 2500 rpm for 40 s, then the ITO substrates with ZnO/methionine films were transferred into a high-purity nitrogen-filled glove box. The room-temperature PM6:BTP-ec9 blend solution (20 µl) was spin-coated onto the top of the methionine layer at 3800 rpm for 40 s, then the active layers were annealed at 80 °C for 5 min. A thin molybdenum trioxide ($MoO_3$) layer (10 nm) and a silver (Ag) layer (100 nm) were sequentially deposited by thermal evaporation under $10^{-5}$ Pa, and the thickness was monitored by a quartz crystal microbalance. The active area of each device is approximate 3.8 mm$^2$, which is defined by the vertical overlap of the ITO cathode and Ag anode.

### 1.3. Device characterization

The current density–voltage (*J-V*) curves of the OSCs were measured under 1 sun AM 1.5 G illumination (100 mW cm$^{-2}$) by using a Keithley 2400 source meter in a high-purity nitrogen-filled glove box. The AM 1.5 G illumination was provided by an XES-50S2 (SAN-EI Electric Co., Ltd.) solar simulator (AAA class, 40 × 40 mm$^2$ effective irradiated areas). The external quantum efficiency (EQE) spectra of OSCs were measured by using a Zolix Solar Cell Scan 100 in atmospheric air. The absorption spectra and transmission spectra of the films were measured using a PERSEE TU-1900 spectrophotometer. The photoluminescence (PL) spectra were measured by a fluorescence spectrophotometer (FLS 1000). The contact angles of films were characterized by a contact angle meter. The morphology

images of films were characterized by an atomic force microscope (AFM, Horiba Bruker Multimode 8). The work functions (WFs) of films were analyzed by Kelvin probe force microscopy (KPFM, Horiba Bruker Multimode 8). Electrochemical impedance spectroscopy (EIS) was measured by an electrochemical workstation (CHI 760 E) in atmospheric air.

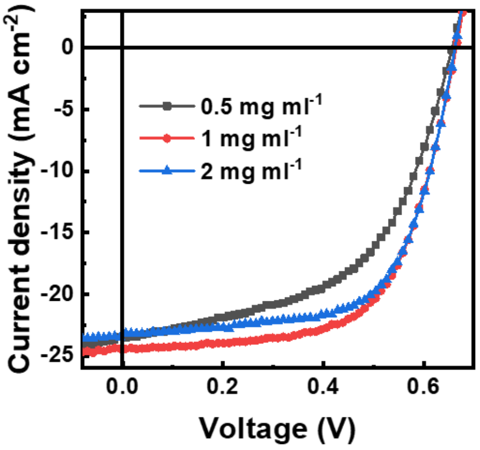


**Figure S1.** *J-V* characteristics curves of OSCs with methionine as ETL under different concentrations of methionine solution.

**Table S1.** Key photovoltaic parameters of OSCs with methionine as ETL under different concentrations of methionine solution.

| Concentration of methionine solution | *Jsc* (mA cm$^{-2}$) | *Voc* (V) | FF (%) | PCE (%) |
|---|---|---|---|---|
| 0.5 mg ml$^{-1}$ | 23.33 | 0.66 | 53.87 | 8.29 |
| 1 mg ml$^{-1}$ | 24.43 | 0.67 | 63.16 | 10.33 |
| 2 mg ml$^{-1}$ | 23.52 | 0.66 | 63.97 | 9.93 |

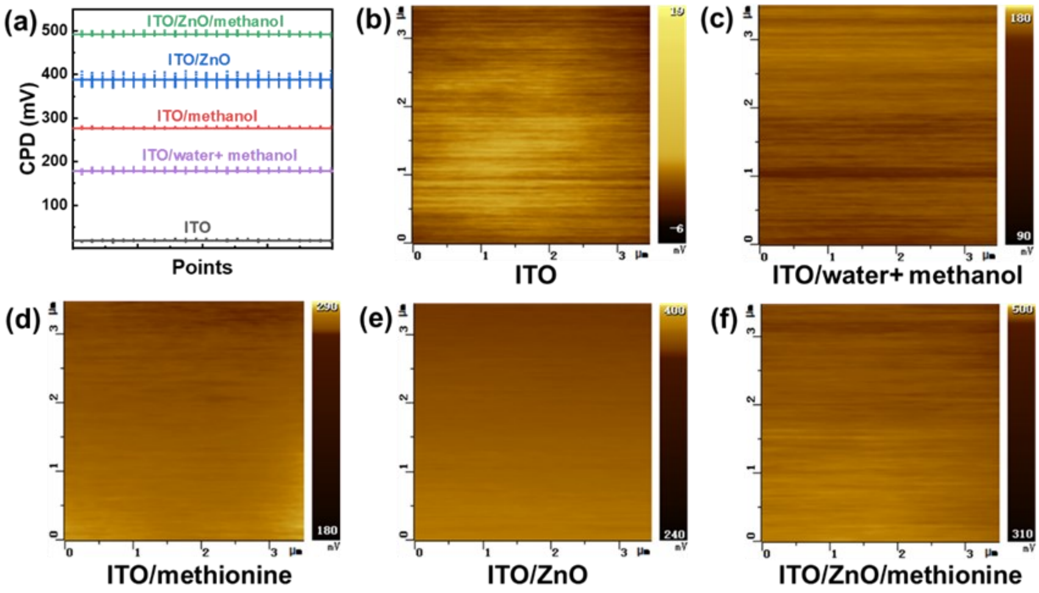


**Figure S2.** (a) CPD data and (b-f) CPD images of ITO modified by different ETLs captured in KPFM.

**Table S2.** CPD and WFs of ITO modified by different ETLs.

| ETLs | CPD (mV) | WF (eV) |
|---|---|---|
| Without | 20 | 4.70 |
| Water+methanol | 181 | 4.54 |
| Methionine solution | 280 | 4.44 |
| ZnO | 393 | 4.33 |
| ZnO/methionine solution | 496 | 4.23 |

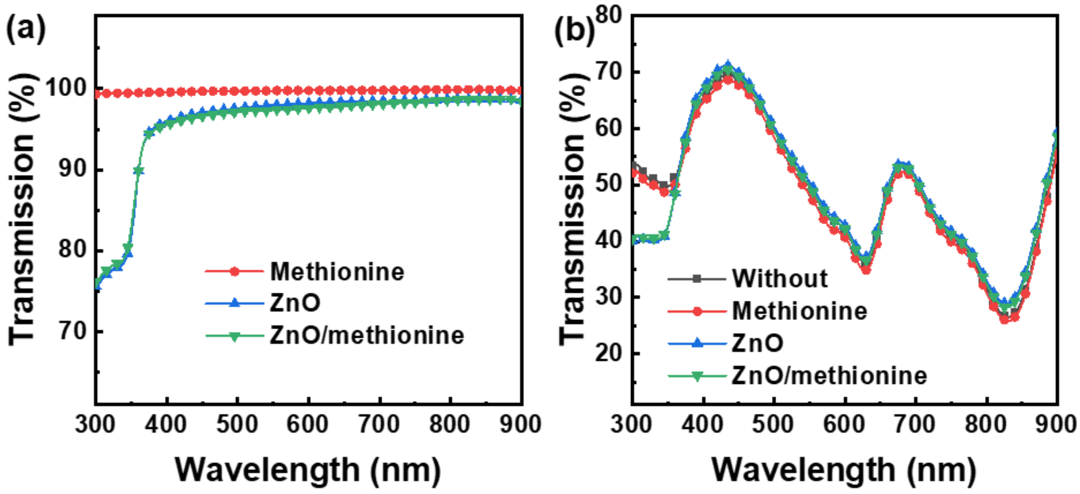


**Figure S3.** (a) Transmission spectra of different ETLs films and (b) different ETLs/active layer films.

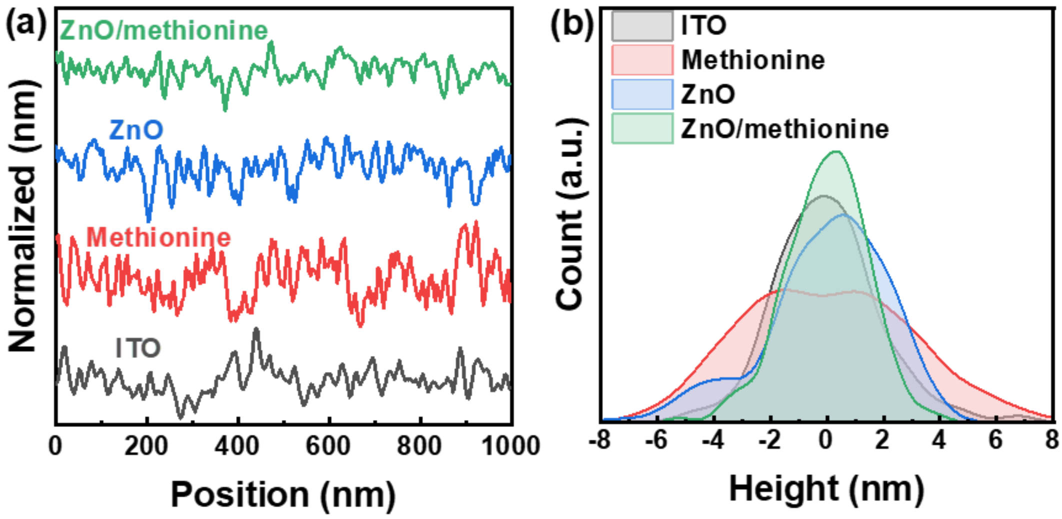


**Figure S4.** (a) Line scanning height of the AFM images of PM6:BTP-ec9 films spin-coated on ITO, ITO/methionine, ITO/ZnO, and ITO/ZnO/methionine; (b) surface height histogram extracted from corresponding AFM image.

The structure of electron-only devices is ITO/ETL/active layer/PDIN/Al. The structure of hole-only devices is ITO/PEDOT:PSS/active layer/$MoO_3$/Ag. The fabrication conditions of the active layer films are the same as those for the OSCs. The charge mobilities are generally described by the Mott-Gurney equation:

$$J = \frac{9}{8}\varepsilon_r\varepsilon_0\mu\frac{V^2}{L^3}$$

where *J* is the current density, $\varepsilon_0$ is the permittivity of free space, $\varepsilon_r$ is the dielectric constant of used materials, $\mu$ is the charge mobility, *V* is the applied voltage, and L is the active layer thickness. In organic materials, charge mobility is usually field-dependent and can be described by the disorder formalism, typically varying with the electric field, E=V/L, according to the equation:

$$\mu = \mu_0 \exp[0.89\gamma\sqrt{\frac{V}{L}}]$$

where $\mu_0$ is the charge mobility at zero electric field and $\gamma$ is a constant. Then, the Mott-Gurney equation can be described by:

$$J = \frac{9}{8}\varepsilon_r \varepsilon_0 \mu_0 \frac{V^2}{L^3}\exp[0.89\gamma\sqrt{\frac{V}{L}}]$$

In this case, the charge mobilities were estimated using the following equation:

$$\ln(\frac{JL^3}{V^2}) = 0.89\gamma\sqrt{\frac{V}{L}} + \ln(\frac{9}{8}\varepsilon_r \varepsilon_0 \mu_0)$$

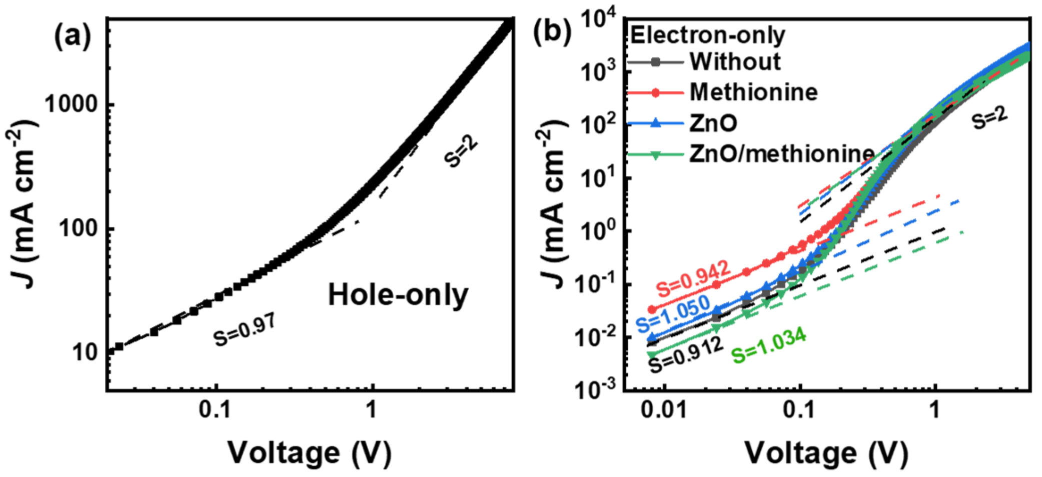


**Figure S5.** (a) Shows a typical plot of log (*J*) versus log (*V*) curves obtained when holes are injected into a hole-only device, and (b) shows the same curve when electrons are injected into a purely electronic device. The dotted line represents the slope.

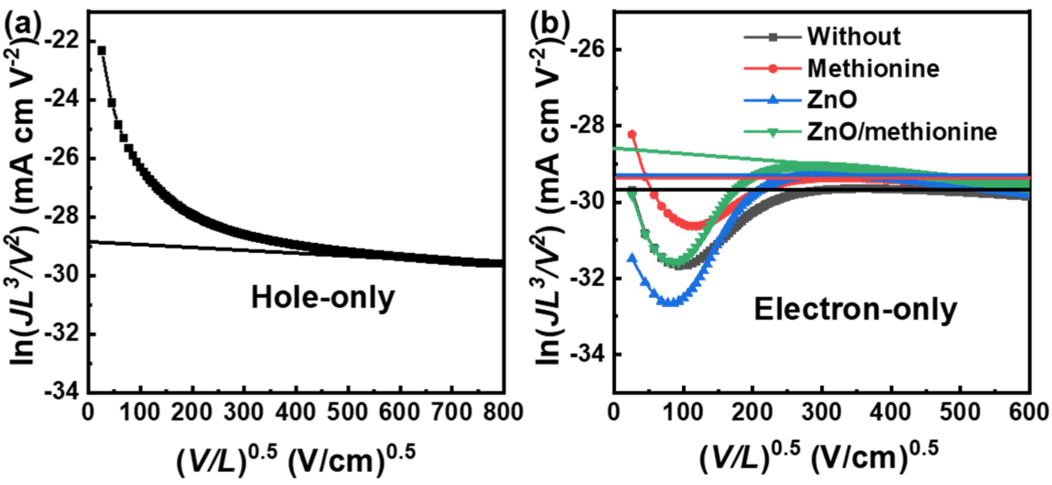


**Figure S6.** $\ln(JL^3/V^2)$-$(V/L)^{0.5}$ curves for the SCLC fitting of (a) hole-only devices with the structure of ITO/PEDOT:PSS/active layer/$MoO_3$/Al and (b) electron-only devices with the structure of ITO/ETL/active layer/PDIN/Al.

**Table S3.** Hole mobility and electron mobility extracted from the SCLC model.

| ITO/ETLs | $\mu_h$ ($cm^2\,V^{-1}\,s^{-1}$) | $\mu_e$ ($cm^2\,V^{-1}\,s^{-1}$) | $\mu_h/\mu_e$ |
|---|---|---|---|
| Without | | $4.69\times10^{-4}$ | 2.18 |
| Methionine solution | | $5.72\times10^{-4}$ | 1.79 |
| ZnO | $1.02\times10^{-3}$ | $6.33\times10^{-4}$ | 1.62 |
| ZnO/methionine solution | | $1.27\times10^{-3}$ | 0.80 |

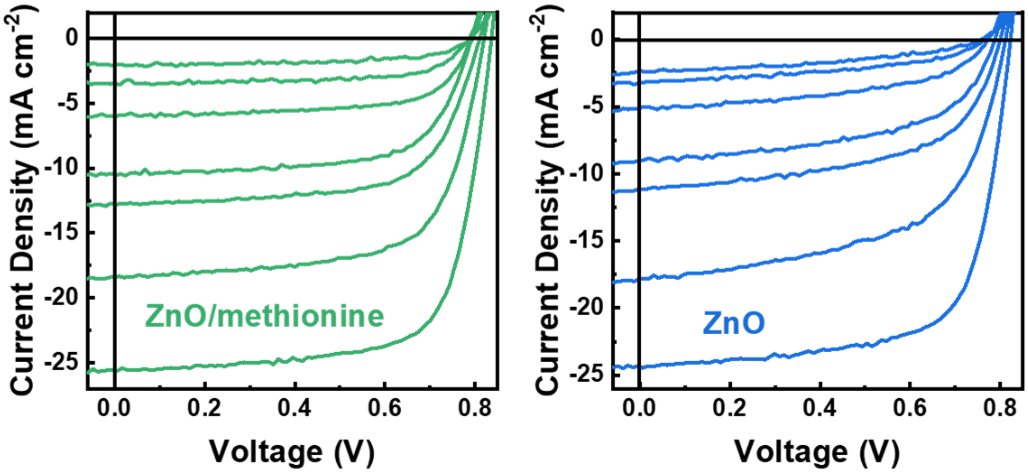


**Figure S7.** *J-V* curves of OSCs with ZnO or ZnO/methionine as ETLs under AM 1.5G illumination with the light intensities of 100, 79, 50, 39.5, 25, 12.5, or 10 mW $cm^{-2}$, respectively.

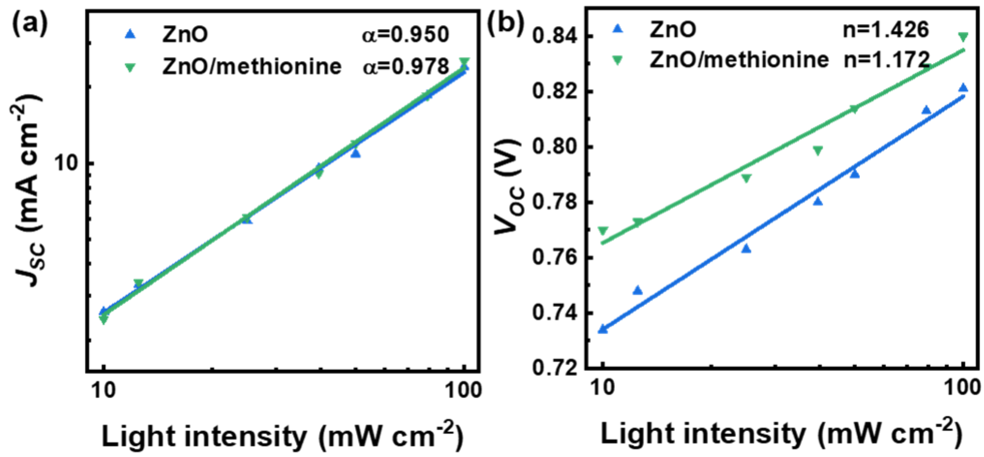


**Figure S8.** (a) $J_{SC}$ and (b) $V_{OC}$ dependence on the light intensity of OSCs with ZnO or ZnO/methionine as ETLs.

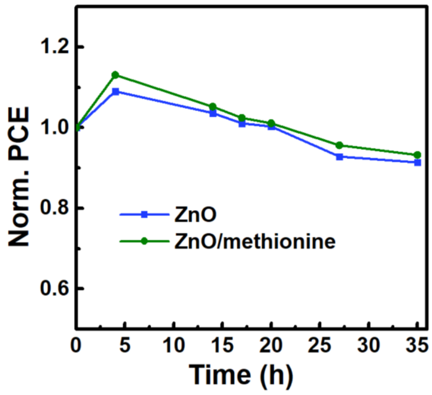


**Figure S9.** Normalized PCE versus storage time for ZnO or ZnO/methionine-based OSCs stored in a high-purity nitrogen-filled glovebox.
